# Prevention of M2 polarization and temporal limitation of differentiation in monocytes by extracellular ATP

**DOI:** 10.1186/s12865-023-00546-3

**Published:** 2023-06-23

**Authors:** Benedikt F. Scherr, Martin F. Reiner, Flavia Baumann, Kerstin Höhne, Tobias Müller, Korcan Ayata, Joachim Müller-Quernheim, Marco Idzko, Gernot Zissel

**Affiliations:** 1 Department of Pneumology, Medical Center, Faculty of Medicine, University of Freiburg, Engesserstr. 4 5thFloor, 79106 79108 Freiburg, Germany; 2 Institute of Intensive Care Medicine, University Hospital Zurich, 8091 Zurich, Switzerland; 3 Department of Cardiology, University Heart Center, University Hospital Zurich, 8091 Zurich, Switzerland; 4 Emergency Department, University Hospital Zurich, 8091 Zurich, Switzerland; 5 Department of Pneumology, University Medical Center Mannheim, University of Heidelberg, 68167 Mannheim, Germany; 6 Department of Biomedicine, University of Basel, 4031 Basel, Switzerland; 7 Division of Pulmonology, Department of Medicine II, Medical University of Vienna, 1090 Vienna, Austria

**Keywords:** Monocyte, ATP, Differentiation, CCL18, M2 macrophage

## Abstract

**Background:**

Elevated levels of extracellular adenosine triphosphate (ATP) modulate immunologic pathways and are considered to be a danger signal in inflammation, lung fibrosis and cancer. Macrophages can be classified into two main types: M1 macrophages are classically activated, pro-inflammatory macrophages, whereas M2 macrophages are alternatively activated, pro-fibrotic macrophages. In this study, we examined the effect of ATP on differentiation of native human monocytes into these macrophage subtypes. We characterized M1 and M2 like macrophages by their release of Interleukin-1beta (IL-1β) and Chemokine (C–C motif) ligand 18 (CCL18), respectively.

**Results:**

Monocytes were stimulated with ATP or the P2X7 receptor agonist Benzoylbenzoyl-ATP (Bz-ATP), and the production of various cytokines was analyzed, with a particular focus on CCL18 and IL-1β, along with the expression of different purinergic receptors. Over a 72 h period of cell culture, monocytes spontaneously differentiated to M2 like macrophages, as indicated by an increased release of CCL18. Immediate stimulation of monocytes with ATP resulted in a dose-dependent reduction in CCL18 release, but had no effect on the concentration of IL-1β. In contrast, delayed stimulation with ATP had no effect on either CCL18 or IL-1β release. Similar results were observed in a model of inflammation using lipopolysaccharide-stimulated human monocytes. Stimulation with the P2X7 receptor agonist Bz-ATP mimicked the effect of ATP on M2-macrophage differentiation, indicating that P2X7 is involved in ATP-induced inhibition of CCL18 release. Indeed, P2X7 was downregulated during spontaneous M2 differentiation, which may partially explain the ineffectiveness of late ATP stimulation of monocytes. However, pre-incubation of monocytes with PPADS, Suramin (unselective P2X- and P2Y-receptor blockers) and KN62 (P2X7-antagonist) failed to reverse the reduction of CCL18 by ATP.

**Conclusions:**

ATP prevents spontaneous differentiation of monocytes into M2-like macrophages in a dose- and time-dependent manner. These effects were not mediated by P2X and P2Y receptors.

**Supplementary Information:**

The online version contains supplementary material available at 10.1186/s12865-023-00546-3.

## Background

Monocytes are leukocytes that originate from CD34^+^ (cluster of differentiation)-progenitor cells in the bone marrow, circulate in the blood stream and migrate to various tissues, where they differentiate to tissue resident macrophages and dendritic cells [1]. The activation and polarization of the heterogeneous mononuclear phagocytic cells can affect immune responses and thus the development of various immune-mediated diseases. Macrophages and dendritic cells play a crucial role in directing the adaptive immune system towards Th1 (T helper 1) or Th2 cell responses. Macrophages contribute to this polarization through diverse signals of M1 and M2 macrophages [2–4]. The functional polarization generating the high plasticity of the mononuclear cells can be described by two different forms of macrophages: the M1 or classically activated macrophages, typically developing after exposure of monocytes to the cytokine IFNγ (interferon gamma) and/ or the bacterial endotoxin LPS (Lipopolysaccharide); and the M2 or alternatively activated macrophages, typically developing after activation via IL-4 (Interleukin-4) and IL-13. Furthermore, M2 macrophages can be further differentiated (M2a – M2d) depending on the causative stimulus [3]. Both M1 and M2 macrophages represent extremes of a continuum caused by different stimuli and subsequent alterations in gene expression [2, 3, 5].

M1 macrophages are generally characterized as potent effector cells producing immunological relevant molecules such as reactive oxygen species and proinflammatory cytokines, including TNFSF2 (Tumor necrosis factor superfamily member 2), IL-1β and IL-6. They are activated by Th1 cells and induce Th1 responses in return. M1 cells exhibit pro-inflammatory and microbicidal characteristics and promote strong IL-12-mediated Th1 responses. Furthermore, they promote anti-tumor immune responses [3].

On the other hand, M2 macrophages synthesize a wide panel of cytokines depending on the activation pathway. In general, they produce high levels of IL-1Ra and CCL18 (Chemokine (C–C motif) ligand), but only a small amount of mature IL-1β [6]. M2 macrophages promote tumor progression [3, 7], tissue repair, and remodeling processes [8, 9]. Additionally, M2 polarized macrophages exhibit Th2-associated anti-inflammatory effector functions characterized by increased synthesis of trophic and pro-fibrotic factors, accompanied by reduced pro-inflammatory cytokine secretion [9].

The M2 subpopulation of macrophages responds to a broad range of activation pathways apart from M1 macrophages, including activation via IL-4 or IL-13 (M2a), immune complexes with IL-1β or LPS (M2b) and IL-10, TGFβ (Transforming growth factor beta; M2c) [2, 3, 7, 9]. However, M2 activation can also be induced by collagen and products released by fibroblasts or tumor cells [8–10].

The production of CCL18 is a typical marker for alternatively activated macrophages [11]; while monocytes stimulated with CCL18 themselves resemble M2 macrophages [12]. Thus, CCL18 plays an important role in the immunopathogenesis of fibrosis [13, 14] and cancer [15–17]. Enhanced CCL18 expression is observed in various diseases ranging from different tumors to hypersensitivity pneumonitis, chronic-obstructive pulmonary disease to fibrotic diseases [8, 9, 13–15, 18–22]. Hence, these diseases are all characterized by differentially polarized macrophages.

Studies have demonstrated the crucial role of extracellular ATP (adenosine triphosphate) in cell-to-cell communication in the immune system of the lung [23, 24]. ATP is an endogenous danger signal released by damaged or stressed cells (damage associated molecular patterns (DAMPS)). However, the concentration of extracellular ATP is tightly regulated through ectonucleotidases, such as CD39 and CD73, which dephosphorylate ATP to ADP (adenosine diphosphate), AMP (adenosine monophosphate), and lastly adenosine, thereby maintaining the ATP concentrations at approximately 10 nM [25, 26]. In contrast, interstitial ATP concentrations in tumor tissues have been detected in the μM range [27]. Extracellular ATP modulates diverse cell functions, altering the electrolyte balance and the shape of cells [28]. However, prolonged stimulation with ATP may lead to apoptosis through DNA (Deoxyribonucleic acid)-fragmentation, condensation of chromatin, blebbing of the cell surface and breakdown of the nucleus [29, 30].

There are multiple different purinergic receptors expressed on monocytes and macrophages. Macrophages primarily express P2Y2-receptors, while P2Y1-, P2Y4-, P2Y11-, and P2Y12-receptors are found on alveolar macrophages and the receptors P2Y1, P2Y2, P2Y4 and P2Y6 on monocytes [31]. Among ion-channels, P2X7 is the primary purinergic receptor subtype found on macrophages and monocytes, apart from P2X1 and P2X4 receptors [32]. Increased ATP levels have been observed in bronchoalveolar lavage fluid from patients with idiopathic pulmonary fibrosis (IPF) and animals with bleomycin-induced pulmonary fibrosis [33, 34]. As ATP has been shown to downregulate TNF and IL-12p40 but increases the release of mature IL-1β [32], all mediators from the M1 side of the macrophage spectrum, the role of ATP in the shift to the M1 or M2 side of the macrophage spectrum is unclear. Of particular interest is CCL18, as increased release of this chemokine by alveolar macrophages and increased concentrations from BAL fluids of IPF patients have been reported [8]. Notably, fewer P2-receptors, such as P2X7- or P2Y2-receptors, were associated with reduced inflammation and fibrosis after bleomycin or silica administration [33–35], but also maturation of the cells influences the expression of ATP receptors [23]. Therefore, in this study, we aim to analyze the role of ATP in the M1/M2 shift of human monocytes, primarily monitored by their CCL18 release, as well as the changes in the expression of purinergic receptors over time.

## Materials and methods

### Isolation and culture of peripheral blood monocytes

Blood samples were collected from 11 healthy human volunteers, and PBMC (peripheral blood mononuclear cells) were isolated by density centrifugation (Ficoll, GE Healthcare, Chalfont St Giles, United Kingdom). The study was conducted according to the guidelines of the Declaration of Helsinki and approved by the Ethics committee of the Medical Center of the University of Freiburg (vote 280/08). Informed consent of the participants was obtained. Monocytes were isolated from PBMC using anti-human CD14 coated magnetic beads (Miltenyi Biotec, Bergisch Gladbach, Germany) and the magnetic cell separation employing a positive selection strategy (MACS, Miltenyi Biotec) according to the manufacturer's protocol. Monocytes were re-suspended and cultured at 1 × 10^6^ cells per ml in RPMI-1640 (GIBCO, Invitrogen by Life Technologies, Paisley, United Kingdom) with 10% FCS (fetal calf serum) and 1% penicillin/streptomycin in 24-well plates at 1 ml in a humidified atmosphere containing 5% CO_2_ at 37 °C for 72 h. After seeding, the monocytes were stimulated either immediately, i.e. at 0 h (early stimulation) or after 48 h (late stimulation) with ATP at different concentrations (10 mM and 100 mM) (Sigma-Aldrich, St. Louis, USA) or with the potent but non-specific P2X7-agonist BzATP (Benzoylbenzoyl-ATP, partial agonist at P2X1 and P2Y1) at concentrations of 10 mM or 100 mM (Sigma-Aldrich). The control group was left untreated. A simultaneous treatment of monocytes with ATP and LPS (Sigma-Aldrich) at a concentration of 1 µg/ml was conducted as a model of inflammation. To block ATP-receptors, pyridoxalphosphate-6-azophenyl-2′,4′-disulphonic acid (PPADS, Tocris Bioscience), 1-(N,O-bis{5-isoquinolinesufonyll}-N-methyl-L-tyrosyl)-4-phenyl-piperazine (KN62, Tocris Bioscience) or Suramin (Tocris Bioscience) were added at indicated concentrations.

### ELISA (Enzyme-linked Immunosorbent Assay) for CCL18 and IL-1β

After 72 h, the supernatants were harvested and stored at -80 °C until quantification of production of CCL18 and IL-1β (as well as IL-1Ra and TNFSF2, supplementary data) using DuoSet ELISA Development System Kits (R&D Systems, Minneapolis, USA) according to the protocols provided by the manufacturer.

### RNA-isolation, reverse transcriptions and real time PCR for purinergic receptors

RNA (Ribonucleic acid) was extracted using TRIzol Reagent according to the supplier's protocol (Invitrogen Corporation, Carlsbad, USA). RNA-contents were determined using a NanoDrop spectrophotometer (Thermo Fisher Scientific, Waltham, USA). Total-RNA was reverse-transcribed with iScript cDNA synthesis kit (Bio-Rad Laboratories, Hercules, USA) including random oligo(dT) primer according to the manufacturer's protocol. Primers for human GAPDH, CCL18, P2Y2, P2Y4, P2Y11, P2Y12 and P2X7 (Table 1) were designed with AmplifX version 1.44 by Nicolas Jullien (http://ifrjr.nord.univ-mrs.fr/AmplifX-Home-page) using sequences from the GenBank database (National Center for Biotechnology Information). Primers for P2Y2 and P2Y12 were designed for the consistent region of all isoforms. All primers were synthesized by biomers.net (biomers.net GmbH, Ulm, Germany). The sequences are depicted in Table 1. Real time PCR (Polymerase chain reaction) was performed with iQ SYBR Green SuperMix according to the supplier’s recommendations using an iCycler thermocycler and iCycler 3.0 software (Bio-Rad Laboratories). Amplification conditions were 15 min of initial denaturation at 95 °C followed by annealing (57 °C) for 15 s and amplification at 72 °C for 20 s and denaturation at 95 °C for 10 s.

**Table 1:** Rt-PCR targets with forward and reverse primers

Target	Forward	Reverse
P2Y2	GCCCCTGGAATGACACCATCAAT	CGTACTTGAAGTCCTCGTTGAAGC
P2Y4	GCTGACTGCCGAGTACTGAACATT	GACGGAGCTGACGTCGATATTTGT
P2Y11	AAGTCCTGCCCTGCCAACTTCT	TTCCGGATGCTGAAGCGGTACA
P2Y12	ATCTGGGCATTCATGTTCTTACTC	TGCCAGACTAGACCGAACTCT
P2X7	AGATCGTGGAGAATGGAGTG	TTCTCGTGGTGTAGTTGTGG
CCL18	CCCTCCTTGTCCTCGTCTG	GCTTCAGGTCGCTGATGTATT
GAPDH	CACCAGGGCTGCTTTTAACT	GATCTCGCTCCTGGAAGATG

Verification of the specificity of the amplified products was determined by melting curve analysis and amplification efficiency was stated by running log dilutions of the respective control cDNA. For each sample, a cycle threshold Ct value was calculated independently in duplicates. Relative expression levels of CCL18, P2Y2, P2Y4, P2Y11, P2Y12 and P2X7 were calculated with the formula 2^(Ct GAPDH – Ct Target)^ × 10.000 for each sample.

### Statistical analysis

**Fig. 1:**
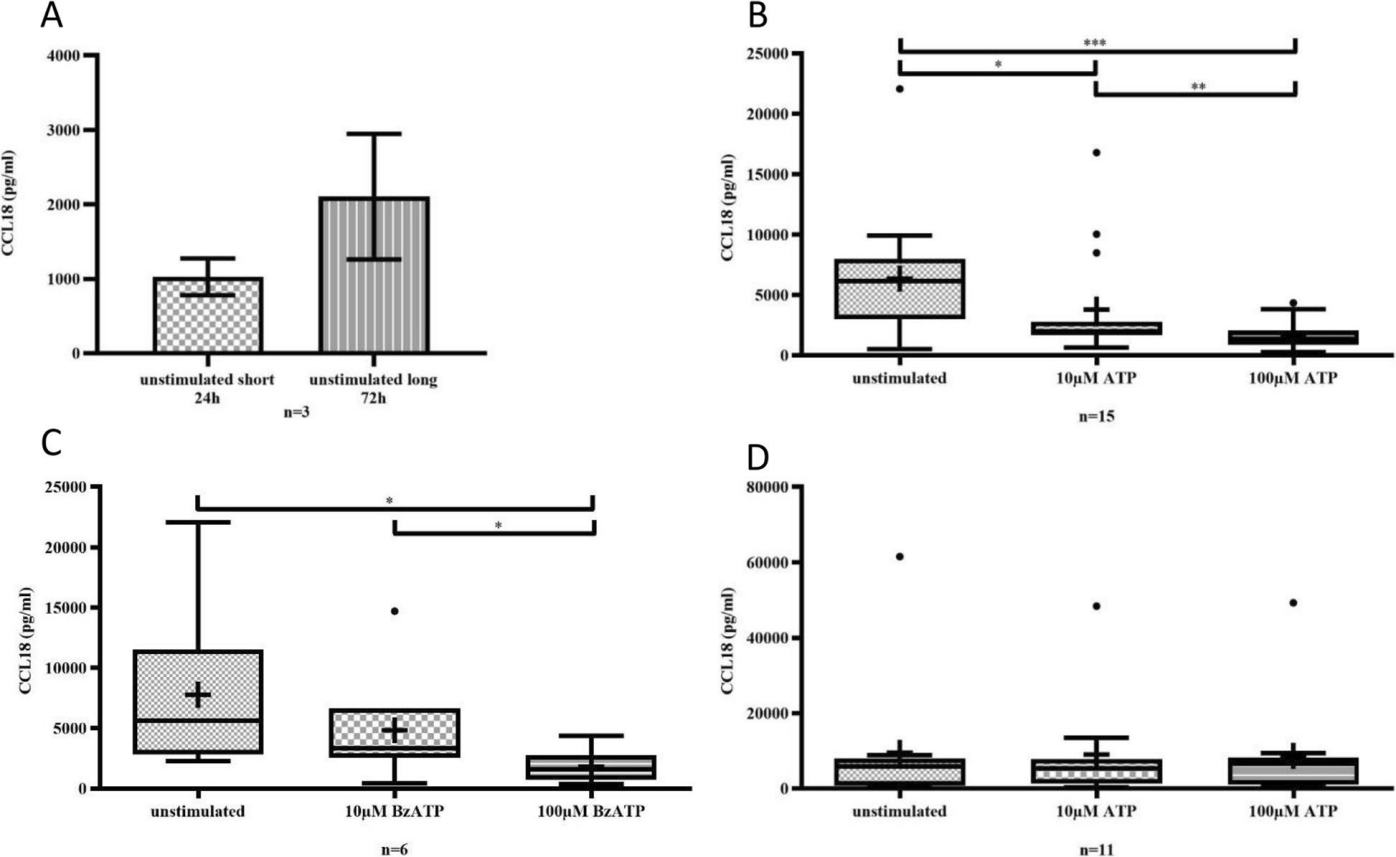
The production of CCL18 increased over time in the cell culture of unstimulated monocytes (A; unstimulated short culture 24 h vs. unstimulated long culture 72 h, n.s.). During the 72-h cell culture period, the levels of CCL18 were reduced in a concentration-dependent manner by immediate stimulation of monocytes with ATP (B; unstimulated vs. 10 µM ATP, *p* = 0.0125; unstimulated vs. 100 µM ATP, *p* = 0.0001). After immediate stimulation with BzATP, the same effect was observed (C). The levels of CCL18 were unaffected when monocytes were stimulated with ATP (D) after 48 h of culture (unstimulated vs. 10 µM ATP, n.s.; unstimulated vs. 100 µM ATP, n.s.)

**Fig. 2:**
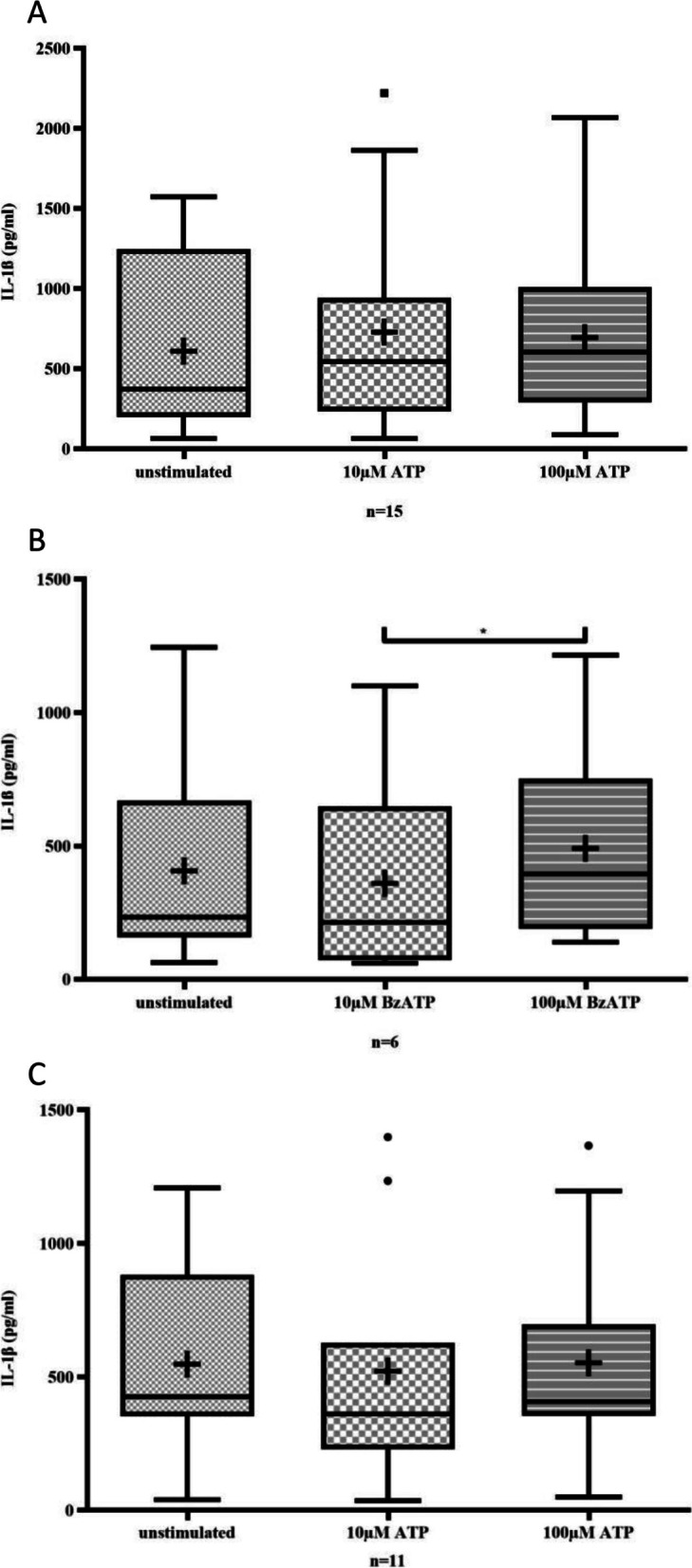
In a 72-h cell culture, neither ATP (A) nor BzATP (B) stimulation increased the levels of IL-1β in monocytes (unstimulated vs. 10 µM ATP, n.s.; unstimulated vs. 100 µM ATP, n.s.; unstimulated vs. 10 µM BzATP, n.s.; unstimulated vs. 100 µM BzATP, n.s.). The levels of IL-1β were not increased in a 72-h cell culture when monocytes were stimulated with ATP (C) after a delay of 48 h (unstimulated vs. 10 µM ATP, n.s.; unstimulated vs. 100 µM ATP, n.s.)

**Fig. 3:**
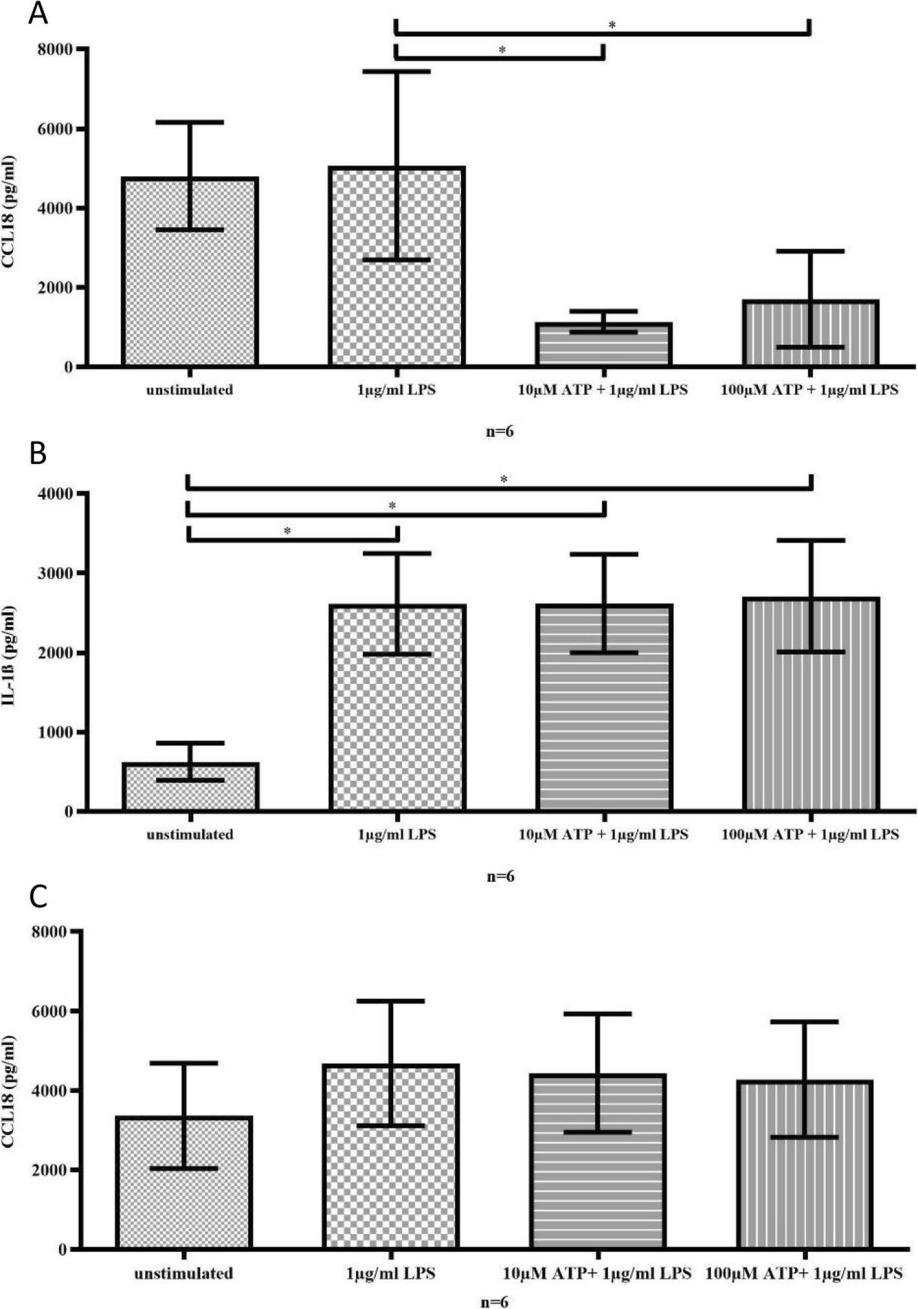
In a model of inflammation that utilized LPS-stimulated monocytes, the early and immediate addition of ATP decreased the levels of CCL18 (A; 1.0 µg/mL LPS vs. 1.0 µg/mL LPS + 10 µM ATP, *p* < 0.05; 1.0 µg/mL LPS vs. 1.0 µg/mL LPS + 100 µM ATP, *p* < 0.05). In contrast, the early addition of ATP to LPS-stimulated monocytes did not alter the levels of IL-1β (B; 1.0 µg/mL LPS vs. 1.0 µg/mL LPS + 10 µM ATP n.s.; 1.0 µg/mL LPS vs. 1.0 µg/mL LPS + 100 µM ATP n.s.). Similarly, delayed incubation with ATP (C) did not affect the levels of CCL18 in LPS-stimulated monocytes (1.0 µg/mL LPS vs. 1.0 µg/mL LPS + 10 µM ATP n.s.; 1.0 µg/mL LPS vs. 1.0 µg/mL LPS + 100 µM

**Fig. 4:**
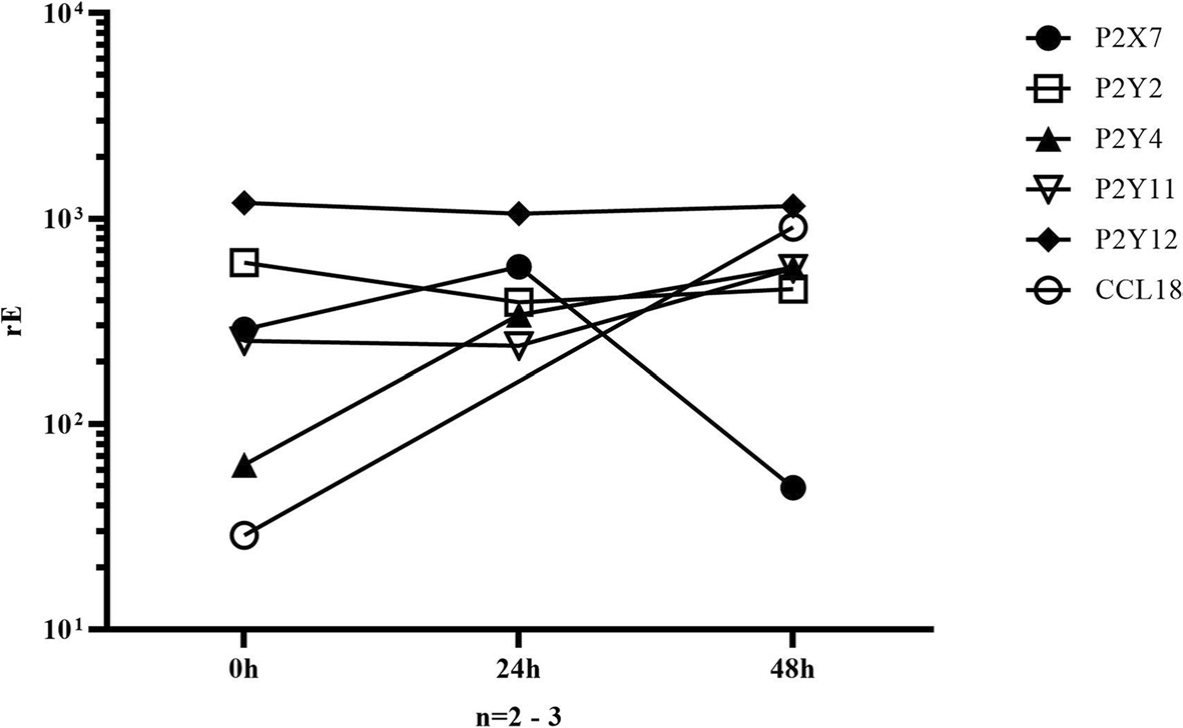
The relative expression (rE) of purinergic receptors P2X7, P2Y2, P2Y4, P2Y11, P2Y12, and CCL18 by monocytes or monocyte-derived macrophages was monitored over time. RNA samples were collected immediately after monocyte isolation and after 24 and 48 h of culture without stimulation. Quantitative rt-PCR was used to analyze the relative expression of the P2 receptors and CCL18 compared to GAPDH (*n* = 2 to 3, depending on the experiment; therefore, significance was not calculated)

**Fig. 5:**
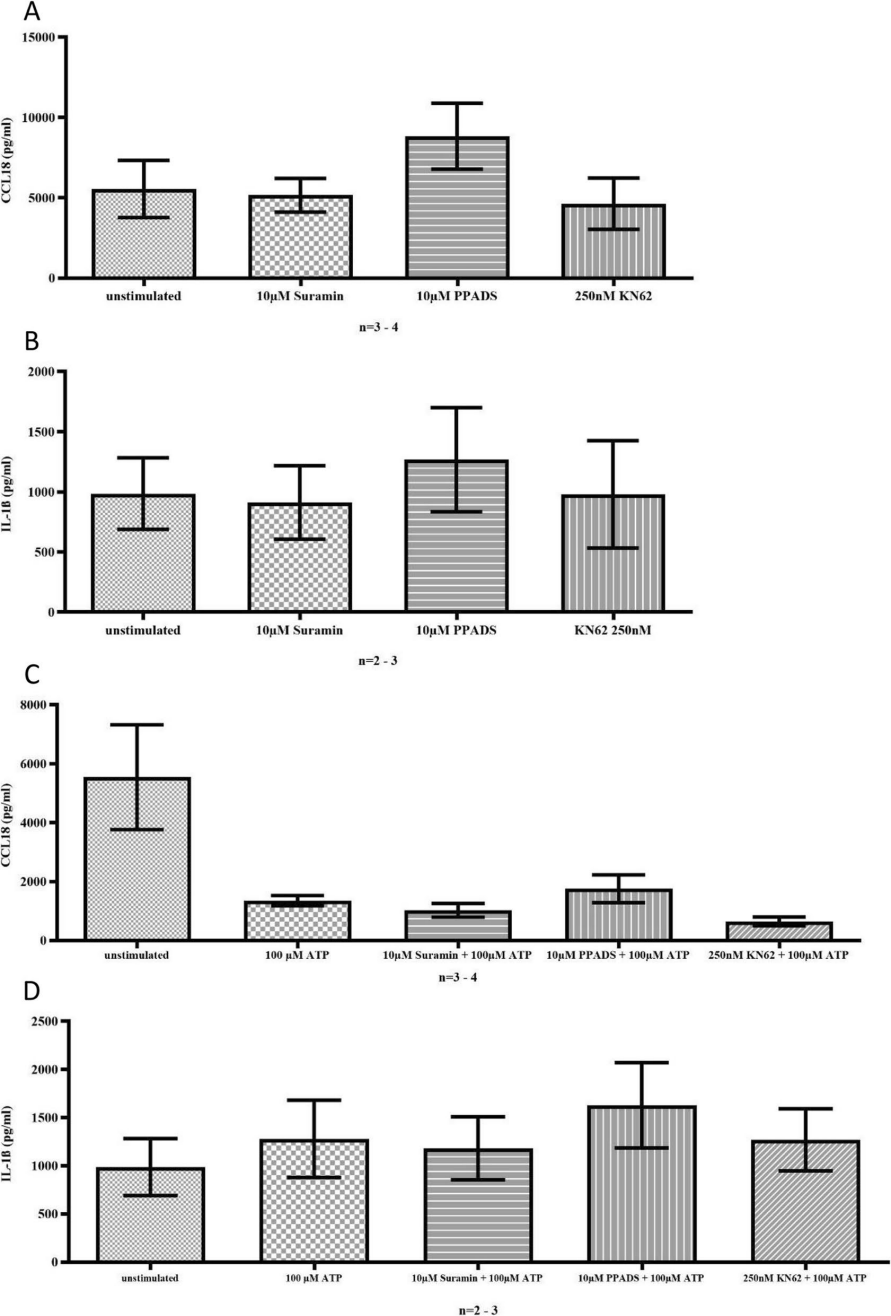
The purinergic antagonists Suramin, PPADS, and KN62 did not have any significant effect on the production of CCL18 (A; unstimulated vs. 10 µM Suramin n.s.; unstimulated vs. 10 µM PPADS n.s.; unstimulated vs. 250 nM KN62 n.s.) or Il-1ß (B; unstimulated vs. 10 µM Suramin n.s.; unstimulated vs. 10 µM PPADS n.s.; unstimulated vs. 250 nM KN62 n.s.). The pre-incubation of monocytes with inhibitors before ATP stimulation did not have any significant effect on the ATP-induced reduction in CCL18 levels (C; 100 µM ATP vs. 10 µM Suramin + 100 µM ATP, *p *= n.s.; 100 µM ATP vs. 10 µM PPADS + 100 µM ATP, *p* = n.s.; 100 µM ATP vs. 250 nM KN62 + 100 µM ATP, *p* = n.s.;) or on IL-1β levels (D; 100 µM ATP vs. 10 µM Suramin + 100 µM ATP, n.s.; 100 µM ATP vs. 10 µM PPADS + 100 µM ATP, n.s.; 100 µM ATP vs. 250 nM KN62 + 100 µM ATP, n.s)

Data are shown as mean ± SEM (Standard error of the mean) in Fig. 1A, 3A-C and 5A-D, as box and whiskers plot (with boxes from 25 to 75th percentiles, Tukey method plot adding 1.5 interquartile range (IQR) as whiskers and outliers as symbols, median as crossbar and mean as cross) in Figs. 1B-D and 2A-C, and in Fig. 4 as mean. Paired Wilcoxon test for comparison of two matched groups and Mann–Whitney test for comparison of independent samples (in case of dropouts) were performed using GraphPad Prism version 5.01/6.01 for Windows (GraphPad Software, San Diego, USA, www.graphpad.com). Differences were considered statistical significant if *p* ≤ 0.05. Significant P values are given asterisks with *p ≤ 0.05, **p ≤ 0.01, ***p ≤ 0.001.

## Results

### ATP inhibited M2 differentiation of monocytes in a concentration- and time-dependent manner

CCL18 secretion increased between 24 and 72 h of cell culture in unstimulated monocytes indicating a spontaneous M2-differentiation over time (Fig. 1A).

When monocytes were stimulated early with ATP (Fig. 1B) or the potent P2X7-agonist BzATP (Fig. 1C), CCL18 secretion showed a dose-dependent decrease. In contrast, when monocytes where stimulated with ATP 48 h after the start of the culture, there was no effect on CCL18 secretion (Fig. 1D), suggesting that early exposure, but not late exposure to ATP prevented macrophage M2 differentiation. These effects are not limited to CCL18 alone as we found comparable results when we investigated IL-1Ra release (Suppl. Figure 1).

### ATP did not induce M1 differentiation of monocytes

Neither early (Fig. 2A), nor late (Fig. 2C) stimulation of monocytes using ATP had an effect on IL-1β secretion, indicating that ATP did not induce M1 differentiation of monocytes. Similarly, early stimulation of monocytes with BzATP did not increase IL-1β, suggesting no effect of BzATP on M1 polarization (Fig. 2B). Comparable results are seen regarding TNFSF2 (Suppl. Figure 2).

### ATP prevented M2 differentiation without affecting M1 differentiation during inflammation in a time-dependent manner

In a model of inflammation, the immediate addition of ATP to LPS-stimulated monocytes led to a reduction in CCL18 secretion (Fig. 3A). However, secretion of IL-1β remained non-affected by ATP (Fig. 3B), indicating that ATP prevented M2 differentiation but did not affect M1 differentiation during inflammation.

Again, when ATP was added to LPS-stimulated monocytes delayed after 48 h, we did not observe any effect on CCL18 secretion (Fig. 3C), suggesting that only early but not late exposure to ATP prevented macrophage M2 differentiation in inflammatory conditions.

### The P2X7-receptor is downregulated during differentiation to CCL18-releasing M2-like macrophages

Since ATP affected CCL18 release of monocytes only after immediate and not delayed stimulation and since the P2X7 receptor agonist BzATP replicated this effect, we examined the expression of P2X7 and of G-protein coupled P2Y-receptors (P2Y2, P2Y4, P2Y11, P2Y12) in monocytes over time. Indeed, our findings revealed that the expression of P2X7 decreased over time (Fig. 4), which could explain the time-dependent effect of ATP on monocyte differentiation. Additionally, we noted that the expression of the purinergic receptors P2Y2, P2Y4, P2Y11 and P2Y12 remained stable or increased over time.

### The effect of ATP on M2 differentiation cannot be blocked by known P2X and P2Y receptor antagonists

The addition of Suramin (an inhibitor of P2Y and, to a lesser extent, P2X receptors), PPADS (an inhibitor of P2X1-3, P2X5, P2Y2, and P2Y4), or KN62 (a non-competitive P2X7 antagonist) did not affect CCL18 or IL-1β secretion in unstimulated monocytes (Fig. 5A-B). Pre-incubation of monocytes with these inhibitors for 30 min before stimulation with ATP did not reverse the ATP-induced reduction of CCL18 (Fig. 5C), suggesting that the observed effect of ATP on CCL18 cannot be blocked by known P2X and P2Y receptor antagonistss. Similarly, pre-incubation with P2X and P2Y inhibitors PPADS, Suramin, and KN62 before ATP stimulation had no effect on IL-1β secretion (Fig. 5D).

## Discussion

We employ the paradigm that production of CCL18 as well as Interleukin-1 receptor antagonist (IL-1Ra, supplementary data Fig. 1A, B) on one side, and IL-1β and TNFSF2 (supplementary data Fig. 2A, B) on the other, may reflect the extreme endpoints of the macrophage activation pattern between M1 and M2 macrophages. Unstimulated monocytes cultured for 24 h do not release CCL18, but high levels of IL-RA [9]. Culture of these cells for 72 h induces high levels of CCL18 and IL-1RA, indicating an M2 shift. Interestingly, early stimulation with the danger signal ATP prevented M2 differentiation of monocytes, seen in a consistent and dose-dependent drop in CCL18 levels. On the other hand, IL-1β production was low, possibly induced by danger signals released by dying cells in the culture. Addition of ATP did not induce or fortify further IL-1β release, which would be indicative for an M1 shift. This might be due to the well-known effect that ATP alone, without co-stimulation through LPS, has no significant effect on IL-1β secretion [36–38]. In addition, it has been shown that monocytes may produce ATP themselves, which is sufficient to activate IL-1β maturation and does not require external ATP [39].

In a model of inflammation using LPS as a pro-inflammatory stimulus, early stimulation with ATP prevented M2 differentiation without affecting M1 differentiation. In this case, LPS alone showed only minimal effect on CCL18 release. The impact of LPS on CCL18 release has been discussed before for alveolar macrophages and monocytes. Kollert et al. demonstrated that LPS downregulated CCL18 release in smokers in alveolar macrophages derived from bronchoalveolar lavage [40], but not in non-smokers. A similar, marginal impact of LPS on the secretion of CCL18 was described before in vitro in monocyte-derived dendritic cells [41]. In our study, ATP in combination with LPS profoundly downregulated CCL18 release and inhibited M2-polarization. LPS alone lead to a significant release of IL-1β with no additional effect of the co-stimulation with ATP. Previous studies showed that ATP alone had no effect on the accumulation of intracellular pro-IL-1β without stimulation with LPS [38, 42]. Monocytes seem to be able to release IL-1β in response to LPS alone without additional external ATP and without P2X7 receptor activation [43], whereas macrophages seem to require a secondary stimulus like ATP [44]. Ferrari et al. demonstrated in their pioneering study that the levels of IL-1β produced by macrophages after stimulation with LPS alone were lower than after co-stimulation with LPS and ATP (with a peak at 1 mM ATP) and that this effect was mediated by P2X7 receptors through different mechanisms [37, 38]. Moreover, different subsets of monocytes show different patterns of IL-1β secretion after stimulation with LPS, with higher levels of IL-1β observed after LPS stimulation of classical monocytes compared to the non-classical subset, in both an ATP-dependent and -independent manner, although P2X7 activity was comparable [45].

The classic model of production of IL-1β suggests that cells releasing IL-1β require the activity of Caspase-containing multi-protein complexes (inflammasomes) to cleave pro-IL-1β to IL-1β. According to this model, cells require a Toll-like receptor- (for example LPS) or cytokine-signal for the priming of their inflammasomes, and afterwards an additional activating signal (like ATP) [46]. Vice versa, monocytes can release IL-1β independently of P2X7 via Toll-like-receptor (TLR)-stimulation with LPS [43]. This is likely due to the fact that monocytes are able to release ATP themselves after stimulation of their pathogen-sensing receptors, thereby inducing IL-1β secretion in an autocrine way [39].

Our findings demonstrate that native monocytes possess the intrinsic ability to differentiate without further stimulation into CCL18-releasing M2-like macrophages, as evidenced by the increase in CCL18 levels over time. The presence of extracellular ATP significantly diminished this ability.

The discrepancy observed between high extracellular ATP levels and the predominance of M2 macrophages described in tumor and lung fibrosis [27, 34, 47] could be explained by the fact that intrinsically differentiated M2 macrophages migrate to these tissues. These macrophages and monocytes that have developed to M2 like macrophages without ATP stimulation, would be inert to subsequent ATP stimulation, as our results demonstrate a temporal limitation of differentiation.

The shift preventing a M2 activation pattern is time dependent. ATP needs to be present early at the initiation of the culture. Addition of ATP at later time points (such as at 48 h), when in-vitro differentiation of macrophages has already been initiated or even established, has no effect on the release of CCL18 and IL-1β.

Immune cells express a large variety of purinergic P2X and P2Y receptors. P2Y2-receptors are the predominant subtype in macrophages, although previous investigations showed that alveolar macrophages also express P2Y1-, P2Y4, P2Y11-, and P2Y12-receptors and monocytes express the receptors P2Y1, P2Y2, P2Y4 and P2Y6 [31]. Regarding ligand-gated ion-channels, P2X7 is the most important subtype in macrophages and monocytes alongside P2X1 and P2X4 [32, 48]. Both ATP and the potent P2X7 agonist BzATP disclose a similar pattern of down-regulation of CCL18, indicating that P2X7 might play a critical role in the shift of macrophage differentiation. However, it is worth noting that BzATP is not a specific agonist of the P2X7 receptor, but instead, BzATP is a P2X7 receptor agonist with 5—tenfold greater potency than ATP [49–51]. ATP activation of the P2X7 receptor leads to recruitment and activation of pro-inflammatory cells, and production of pro-inflammatory cytokine IL-1β and inflammatory mediators [35, 46, 52].

The expression of P2X7 receptors in monocytes is positively regulated by the proinflammatory cytokines TNFSF2 and IFNγ, as well as LPS [53]. We have demonstrated decreasing CCL18 levels with increasing concentrations of ATP. A comparable reduction on proinflammatory and antifibrotic cytokines like CCL2 and CCL3 has been observed in dendritic cells, with involvement of the receptors P2Y1 and P2Y11 [54]. Non-selective antagonists PPADS and Suramin (which block most P2X and P2Y receptors) as well as KN62 (which is a P2X7-antagonist) were not able to reverse the ATP-induced reduction of CCL18 release. The early ATP-induced decrease in CCL18 release, and thus the prevention of spontaneous M2 differentiation in cultured monocytes, appears to be independent of P2X and P2Y receptors, as the levels of CCL18 and IL-1β remained unchanged after pre-incubation with these antagonists of purinergic receptors.

However, the ineffectiveness of Suramin and PPADS may be attributed to the mechanism that ATP exerts its effects through P2X7-receptor-activation, since these antagonists have no effect on P2X7. On the other hand, the reason for the ineffectiveness of the specific potent P2X7-inhibitor KN62 is not entirely understood. Our data suggest that KN62 did not affect the down-regulation of CCL18 release induced by ATP. It should be noted that KN62 is not a specific P2X7 receptor antagonist, but its affinity to P2X7 is 5 to 30 times greater than to other receptors [49–51]. Besides, KN62 has multiple pleiotropic effects. For example, KN62 also binds to the Calmodulin-dependent protein-kinase CaMK II and inhibits its activation with a IC50 of 0.9 µM. CaMK II is thought to be involved in M1 activation [55], and its inhibition has been shown to reduce CCL3-release [56]. Thus, it is conceivable that CaMK II inhibition by KN62 might promote M2 activation, resulting in an unchanged CCL18 release.

Despite this, P2X7 receptors seem to play an important role in the M2/M1 polarization since P2X7 was considerably downregulated during the in-vitro generation of macrophages, whereas the other P2-receptors analyzed remained stable. The shift towards an anti-inflammatory phenotype has been reported before not to be due to a loss of P2X7, but rather due to an uncoupling of the receptor from the activation of the inflammasome in macrophages that are already shifted towards a M2 phenotype [57]. The expression of P2X7 is upregulated by proinflammatory cytokines TNFSF2, IFNγ and by LPS and downregulated by stimulation with TGFβ and cAMP [53, 58]. This suggests that P2X7-receptors are elevated in M1 subsets and reduced in the M2 subpopulation. Our findings suggest that the loss of P2X7 leads to anti-inflammatory and pro-fibrotic M2 macrophages, which fits into this picture.

A comparable finding to our observation, in which a loss of P2X7 was observed, has been reported in a study, where a monocyte-/macrophage-like phenotype was induced in HL60 promyelocytes to determine expression of purinergic receptors [59]. The study showed a tenfold downregulation of P2X7 at 24 h after monocytic differentiation, which was followed by a marked loss of P2X7 expression after 48 h.

## Conclusions

In summary, we conclude based on our data that the danger signal ATP prevents the M2 differentiation of monocytes. This applies only for early stimulation, but not for late stimulation with ATP, as monocytes can still develop into a M2 pattern without stimulation. Stimulation with ATP does not induce a M1 differentiation of monocytes. Early stimulation with ATP also inhibits the development of a M2 pattern and does not induce a M1 transformation in an inflammation model with pre-incubation with LPS. Known inhibitors of P2X and P2Y receptors are unable to block the interruption of M2 differentiation by ATP, but it is likely that the receptor P2X7 is lost during the process of M2 differentiation and that this loss is responsible for the ATP irresponsiveness of the generated M2 macrophages. Therefore, ATP plays a pivotal role in influencing the cytokine-milieu produced by monocytes and maturing macrophages and prevents a M2 differentiation characterized by low CCL18 and unchanged IL-1β release.

## Supplementary Information

**Additional file 1: Suppl. Figure 1.** Immediate stimulation of monocytes with ATP (**A**) resulted in a concentration-dependent reduction in IL-Ra levels over a 72-hour cell culture period (unstimulated vs. 10 µM ATP, n.s.; unstimulated vs. 100 µM ATP, n.s.). A similar effect was observed after immediate stimulation with BzATP **B**. Delayed stimulation of monocytes with ATP (**C**), starting after 48 hours of culture, did not have any effect on IL-1Ra levels (unstimulated vs. 10 µM ATP, n.s.; unstimulated vs. 100 µM ATP, n.s.). **Suppl. Figure 2.** Stimulation of monocytes with either ATP (**A**) or BzATP (**B**) did not result in any increase in TNFSF2 levels in a 72-hour culture (unstimulated vs. 10 µM ATP, n.s.; unstimulated vs. 100 µM ATP, n.s.; unstimulated vs. 10 µM BzATP, n.s.; unstimulated vs. 100 µM BzATP, n.s.). Delayed stimulation of monocytes with ATP (**C**) after 48 hours of cell culture did not lead to an increase in TNFSF2 levels in a 72-hour culture (unstimulated vs. 10 µM ATP, n.s.; unstimulated vs. 100 µM ATP, n.s.).

## Data Availability

The datasets used and/or analysed during the current study are available from the corresponding author on reasonable request.

## Abbreviations

ADP: Adenosine diphosphate
AMP: Adenosine monophosphate
ATP: Adenosine triphosphate
BAL: Bronchoalveolar lavage
Bz-ATP: Benzoylbenzoyl-ATP
CaMK: Calmodulin-dependent protein kinase
cAMP: Cyclic adenosine monophosphate
CCL: Chemokine (C–C motif) ligand
CD: Cluster of differentiation
DNA: Deoxyribonucleic acid
cDNA: Complementary deoxyribonucleic acid
CO_2_: Carbon dioxide
Ct: Cycle threshold
DAMPS: Damage associated molecular patterns
ELISA: Enzyme-linked immunosorbent assay
FCS: Fetal calf serum
GAPDH: Glyceraldehyde-3-phophate dehydrogenase
h: Hours
IC50: Half maximal inhibitory concentration
IFNγ: Interferon gamma
IL: Interleukin
IL-1Ra: Interleukin-1 receptor antagonist
IPF: Idiopathic pulmonary fibrosis
IQR: Interquartile range
KN62: 1-(N,O-bis{5-isoquinolinesufonyll}-N-methyl-L-tyrosyl)-4-phenyl-piperazine
LPS: Lipopolysaccharide
M1/2: M1/2 macrophage subpopulation
MACS: Magnetic cell separation
mM: Milimolar
μM: Micromolar
nM: Nanomolar
P2X: Ligand-gated ion channel purinoceptor
P2Y: G protein-coupled puringeric receptor
PBMC: Peripheral blood mononuclear cells
PCR: Polymerase chain reaction
PPADS: Pyridoxalphosphate-6-azophenyl-2′,4′-disulphonic acid
RNA: Ribonucleic acid
RPMI: Roswell Park Memorial Institute medium
rtPCR: Real time polymerase chain reaction
SEM: Standard error of the mean
TGF: Transforming growth factor
Th1/2: T helper 1/2
TLR: Toll-like-receptor
TNFSF: Tumor necrosis factor superfamily member
