# Supplementary material for: Prevention of M2 polarization and temporal limitation of differentiation in monocytes by extracellular ATP
Source: BMC Immunol. 2023 Jun 23;24:11. doi: 10.1186/s12865-023-00546-3 (PMC10288684; doi:10.1186/s12865-023-00546-3)
Supplement: Supplementary file 1 — Additional file 1: Suppl. Figure 1. Immediate stimulation of monocytes with ATP (A) resulted in a concentration-dependent reduction in IL-Ra levels over a 72-hour cell culture period (unstimulated vs. 10 µM ATP, n.s.; unstimulated vs. 100 µM ATP, n.s.). A similar effect was observed after immediate stimulation with BzATP B. Delayed stimulation of monocytes with ATP (C), starting after 48 hours of culture, did not have any effect on IL-1Ra levels (unstimulated vs. 10 µM ATP, n.s.; unstimulated vs. 100 µM ATP, n.s.). Suppl. Figure 2. Stimulation of monocytes with either ATP (A) or BzATP (B) did not result in any increase in TNFSF2 levels in a 72-hour culture (unstimulated vs. 10 µM ATP, n.s.; unstimulated vs. 100 µM ATP, n.s.; unstimulated vs. 10 µM BzATP, n.s.; unstimulated vs. 100 µM BzATP, n.s.). Delayed stimulation of monocytes with ATP (C) after 48 hours of cell culture did not lead to an increase in TNFSF2 levels in a 72-hour culture (unstimulated vs. 10 µM ATP, n.s.; unstimulated vs. 100 µM ATP, n.s.). [file 12865_2023_546_MOESM1_ESM.docx]

Supplementary Data - Results

*1. ATP inhibited M2 differentiation of monocytes in a concentration- and time-dependent manner and the effect of IL-1Ra is comparable to CCL18*

Early stimulation of monocytes with ATP (Suppl. Fig. 1A) or the potent P2X7-agonist BzATP (Suppl. Fig. 1B) showed a trend towards a dose dependent reduction of IL-1Ra secretion. In contrast, ATP stimulation of monocytes 48 h after the start of the culture did not affect IL-1Ra secretion (Suppl. Fig. 1C) suggesting that early but not late exposition to ATP prevented macrophage M2 differentiation.


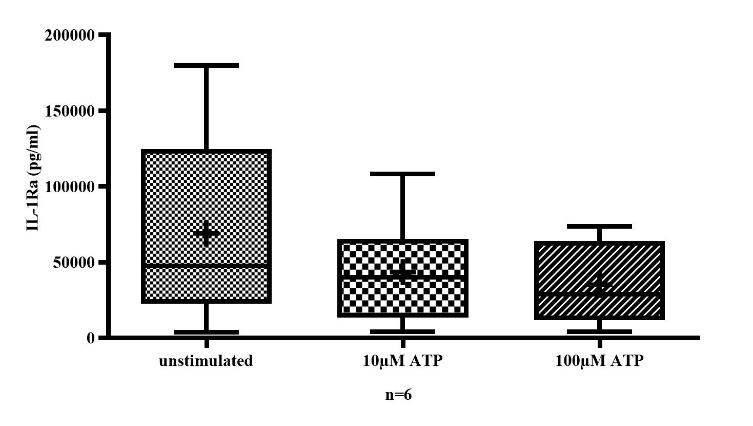

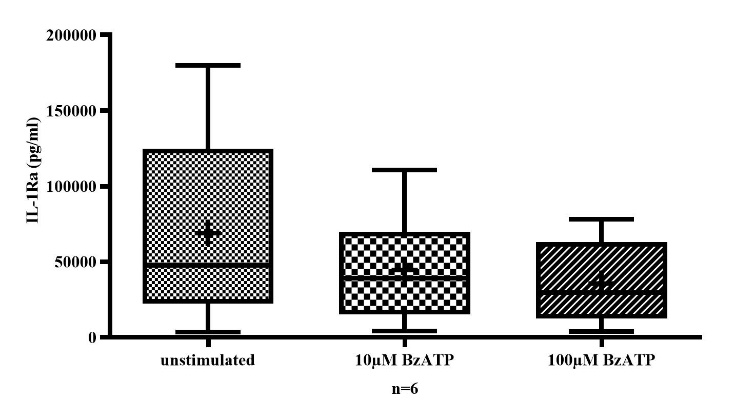

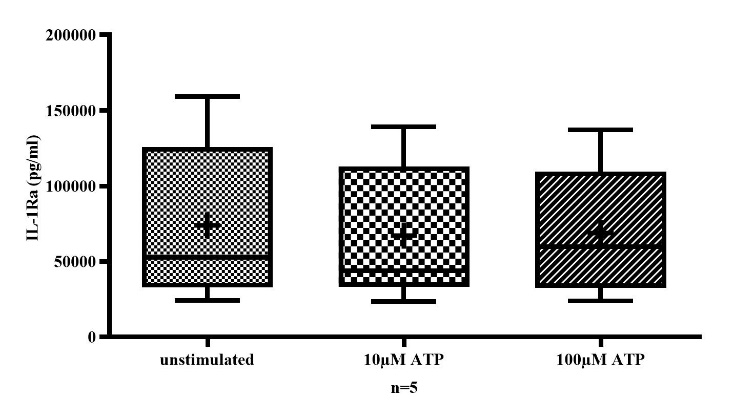


Suppl. Fig. 1A

Suppl. Fig. 1B

Suppl. Fig. 1C

Suppl. Figure 1: Immediate stimulation of monocytes with ATP (A) resulted in a concentration-dependent reduction in IL-Ra levels over a 72-hour cell culture period (unstimulated vs. 10 µM ATP, n.s.; unstimulated vs. 100 µM ATP, n.s.). A similar effect was observed after immediate stimulation with BzATP (B). Delayed stimulation of monocytes with ATP (C), starting after 48 hours of culture, did not have any effect on IL-1Ra levels (unstimulated vs. 10 µM ATP, n.s.; unstimulated vs. 100 µM ATP, n.s.).

*2. ATP did not induce M1 differentiation of monocytes and the effect of TNFSF2 is comparable to IL-1**β*

Neither early (Fig. 2A), nor late (Fig. 2C) stimulation of monocytes using ATP affected TNFSF2 secretion suggesting that ATP did not induce M1differentiation of monocytes. Likewise, early stimulation of monocytes with BzATP did not increase TNFSF2, suggesting no effect of BzATP on M1 polarization (Fig. 2B).


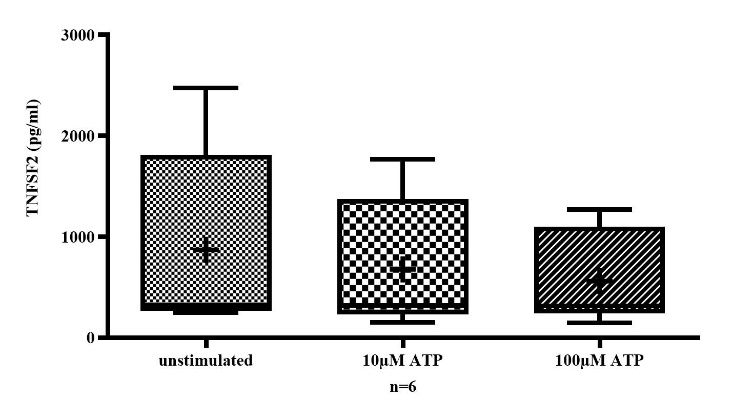

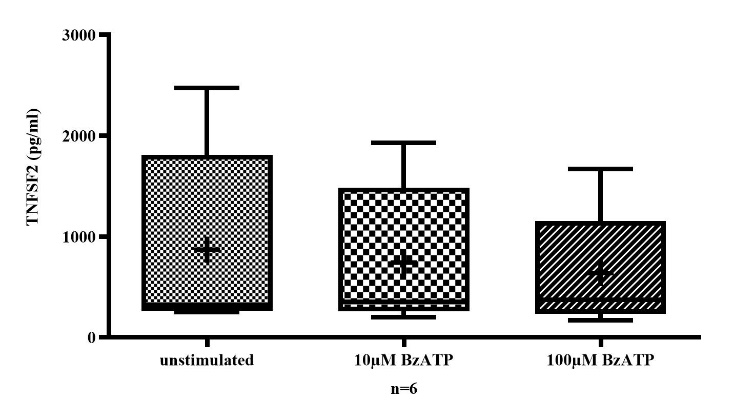

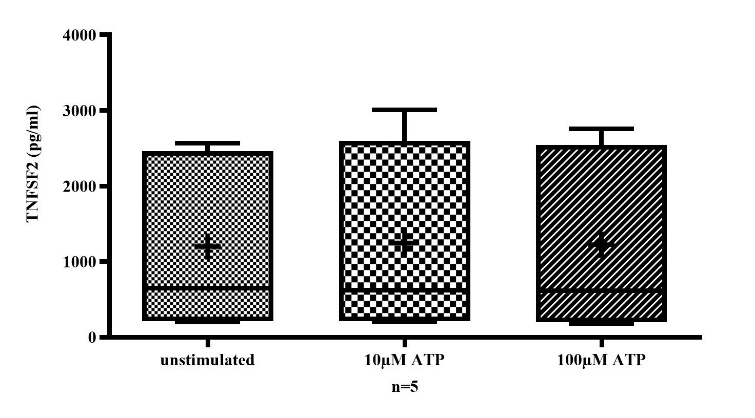


Suppl. Fig. 2A

Suppl. Fig. 2B

Suppl. Fig. 2C

Suppl. Figure 2: Stimulation of monocytes with either ATP (A) or BzATP (B) did not result in any increase in TNFSF2 levels in a 72-hour culture (unstimulated vs. 10 µM ATP, n.s.; unstimulated vs. 100 µM ATP , n.s.; unstimulated vs. 10 µM BzATP, n.s.; unstimulated vs. 100 µM BzATP, n.s.). Delayed stimulation of monocytes with ATP (C) after 48 hours of cell culture did not lead to an increase in TNFSF2 levels in a 72-hour culture (unstimulated vs. 10 µM ATP, n.s.; unstimulated vs. 100 µM ATP, n.s.).
